# Mathematical model of sediment and solute transport along slope land in different rainfall pattern conditions

**DOI:** 10.1038/srep44082

**Published:** 2017-03-08

**Authors:** Wanghai Tao, Junhu Wu, Quanjiu Wang

**Affiliations:** 1 State Key Laboratory Base of Eco-hydraulic Engineering in Arid Area (Xi’an University of Technology), Xi’an, 710048, China; 2 State Key Laboratory of Soil Erosion and Dryland Farming on the Loess Plateau, Institute of Soil and Water Conservation, Northwest A&F University, Xi’an, 712100, China

## Abstract

Rainfall erosion is a major cause of inducing soil degradation, and rainfall patterns have a significant influence on the process of sediment yield and nutrient loss. The mathematical models developed in this study were used to simulate the sediment and nutrient loss in surface runoff. Four rainfall patterns, each with a different rainfall intensity variation, were applied during the simulated rainfall experiments. These patterns were designated as: uniform-type, increasing-type, increasing- decreasing -type and decreasing-type. The results revealed that changes in the rainfall intensity can have an appreciable impact on the process of runoff generation, but only a slight effect on the total amount of runoff generated. Variations in the rainfall intensity in a rainfall event not only had a significant effect on the process of sediment yield and nutrient loss, but also the total amount of sediment and nutrient produced, and early high rainfall intensity may lead to the most severe erosion and nutrient loss. In this study, the calculated data concur with the measured values. The model can be used to predict the process of surface runoff, sediment transport and nutrient loss associated with different rainfall patterns.

Soil erosion and nutrient loss are detrimental to agricultural products, food security, and the sustainability of ecosystem services^1^. Crops can only absorb part of the nutrients contained in the soil, and the remaining nutrients may be lost during rainfall^2^. In recent years, the increased using of chemical fertilizer and corresponding nutrient loss from slope farmland owing to agricultural activities have received increasing attention^3^. Several studies have, through artificial and natural rainfall, studied the mechanism of rainfall erosion. These studies revealed that runoff erosion and nutrient loss processes are affected by the topography, soil properties, and rainfall characteristics^4^. The mechanism of erosion, to a certain extent, depends on the characteristics of the soil. For example, in one case, sandy loam experienced greater nutrient loss than silty clay loam, and nutrient loss in the runoff increased with increasing initial soil water content^5^. Other studies have indicated that the concentration of solute in the runoff increases with increasing slope length and gradient^6^. Moore^7^ evaluated the effect of soil crust on the erosion process and found that the crust can reduce the erosivity of the soil. Moreover, Wang^8^ assessed nitrogen, phosphorus, and potassium transport with runoff and found that soil and water losses increased with increasing gradient. Xing^9^ reported that runoff rates and runoff-associated TN loss rates decreased with increasing slope length, whereas sediment and sediment-associated TN losses increased. Majid^10^ found that rain-induced erosion was transport-limited at gentler slopes, whereas at steeper slopes, this erosion was governed by detachment-limited conditions. Reid^11^ indicated that runoff and sediment production varied significantly with vegetation patch type. Rainfall has a significant effect on the soil fertility of the top layer, because nutrient loss and soil erosion occur mainly in the top of the soil^12^. The effect of rainfall characteristics on soil erosion has been extensively investigated. Ran^13^ concluded that rainfall characteristics have a considerable effect on runoff generation and soil erosion. In semi-arid regions, which are characterized by occurrences of low volume (i.e., annual amount) high-intensity rainfall, variations in precipitation patterns may increase local runoff and soil erosion^14^. Flanagan^15^ found that the runoff rate of storms, with maximum intensity occurring in late stages, was greater than that of uniform-intensity storms or storms with maximum intensity occurring during the initial stages; in addition, soil loss from late-peaking storms was greater than that associated with early-peaking storms. Frauenfeld^16^ reported that rainfall intensity patterns had no effect on the total runoff or infiltration, but the total erosion associated with variable rainfall was significantly greater than that associated with uniform rainfall. Parsons^17^ designed five simulated rainstorms, each with a distinct intensity pattern, which all delivered the same total kinetic energy to the soil surface. Although the resulting total runoff was the same, the amount and size distribution of the eroded sediment varied with the pattern.

Rain-induced erosion research began in early 20th century^18^. Zingg^19^ first began to research the relationship between rain-induced soil erosion and land slope and length, then Smith^20^ expanded the relationship to incorporate conservation practices. In the following study, many models were developed to analyze the process of rainfall-induced erosion^21^. The Universal Soil Loss Equation and its revisions are the most popular empirical water erosion model applied in the world^22^,^23^. Crawford^24^ first developed a physical model (Stanford Watershed Model), which capable of modelling the entire hydrologic cycle and the entire watershed, then the modified model (Hydrological Simulation Program Fortran)^25^ which include water quality processes. Physically-based models are normally based on the conservation equations for water mass and sediment yield^26^. The basic equation used to describe detachment and transport processes from surface runoff is the continuity equation for sediment transport^27^, and Rose^28^ determined that the rate of soil detachment by overland flow and the rate of soil detachment by impacting raindrops.

The process of soil-nutrient release into runoff is quite complex. Raindrop strikes, runoff scour, soil erosion, and diffusion all have an effect on this release. Donigian^29^ hypothesized that rainwater mixes with soil and solute in a shallow thin mixing layer located in the soil surface. Ahujia^30^ used ^32^P as a tracer to analyze solute movement on the surface layer of soil, and concluded that the effect of the mixing layer is limited to depths of 2–3 mm. In subsequent work, Ahujia^31^ found that the complete mixing layer is unsuitable for describing soils that have high infiltration capacity, and proposed an incomplete mixing model for describing solute transport in unsaturated soils. In addition, a series of models based on the theory of an incomplete mixing layer has been established by other researchers^32^,^33^,^34^. Chemical transfer from the soil to the surface runoff was attributed to accelerated diffusion, resulting from soil-depth variations in the chemical concentration of the soil. Hence, the conventional convective-dispersion equation could be used to describe solute transport. Wallach^35^ developed a physically-based diffusion and transport model to describe the transfer of chemicals from the soil solution to the surface runoff. Ahuja^36^ developed a convective-dispersion model by comparing the effects of ordinary molecular diffusion and accelerated diffusion on solute transfer from the soil to the runoff. Raindrop splash and diffusion play an important role in solute transport, as revealed by a solute transport model of raindrop splash (based on the soil erosion model), developed by Gao^37^.

Solute transfer to the soil surface runoff and runoff erosion are influenced by rainfall characteristics. However, the influence of rainfall patterns on runoff erosion and nutrient loss has rarely been investigated. Therefore, the objective of this study is to develop a mathematical model that describes runoff erosion and nutrient loss under different rainfall conditions. The paper is structured as follows: the mathematical model was developed, experimental and numerical results were compared and discussed, some conclusions were drawn. It is hoped that information from this study may be useful in conserving water and soil resources.

## Results

### Surface runoff

**Figure 1:**
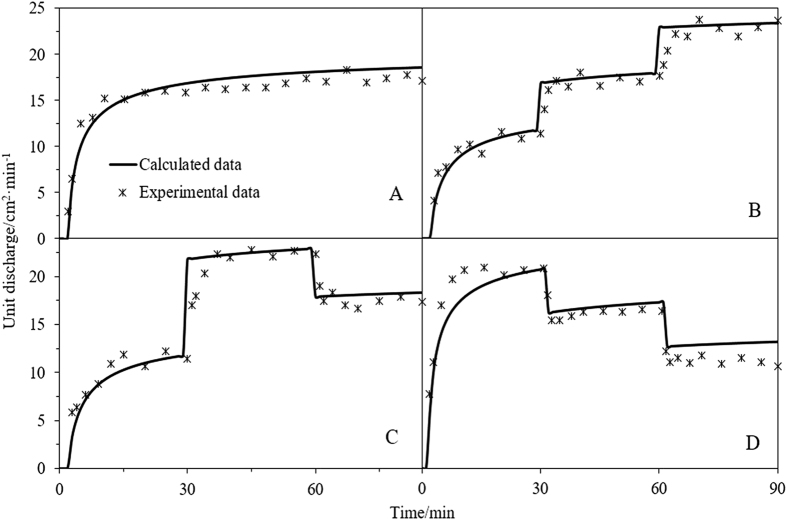
Comparison of the calculated and experimentally determined surface runoff. (**A**) is uniform-type, (**B**) is increasing-type, (**C**) is increasing- decreasing -type and (**D**) is decreasing-type.

The runoff generation associated with each rainfall pattern is shown in Fig. 1. As the figure shows, changes in the rainfall intensity during rainfall can have an appreciable impact on the process of surface runoff. The unit discharge increased sharply and then stabilized at the first stage of rainfall. The main reason of this phenomenon was that the infiltration capacity of soil rapidly decreasing at the beginning of rainfall, then the infiltration rate tends to stable. The rapidly increase or decrease in second and third stages were caused by the change of rainfall intensity. However, for the same rainfall intensity, the unit discharge in the first stage was less than that occurring in the later stages, owing to the higher infiltration capacity of the soil during the early rainfall period. In general, during the entire rain process, almost the same total infiltration volume was obtained for different rainfall patterns. This is evidenced by a total runoff of 143.83, 144.14, 145.08, and 144.91 L, for patterns A, B, C, and D, respectively. The variance analysis showed (see Table 1) that rainfall patterns have no significant influence on total runoff. In other words, changes in the rainfall intensity can have a significant impact on the process of runoff generation, but only a slight effect on the total amount of runoff.

The runoff generation processes (see Fig. 1) were described by Equation (17). Values of the adsorptivity (*S*), constant (*c*), root mean square error (RMSE), and determination coefficient (R^2^) obtained from runoff-generation model fitting are listed in Table 2. The RMSE and R^2^ values reveal the close correspondence between the numerical results and the experimental data. In addition, the value of *c* may decrease with increasing rain intensity, but may also be influenced by the duration of rainfall. In fact, for the same rainfall intensity, the value of *c* associated with the initial stages of rainfall is usually lower than that associated with later periods.

### Sediment

**Figure 2:**
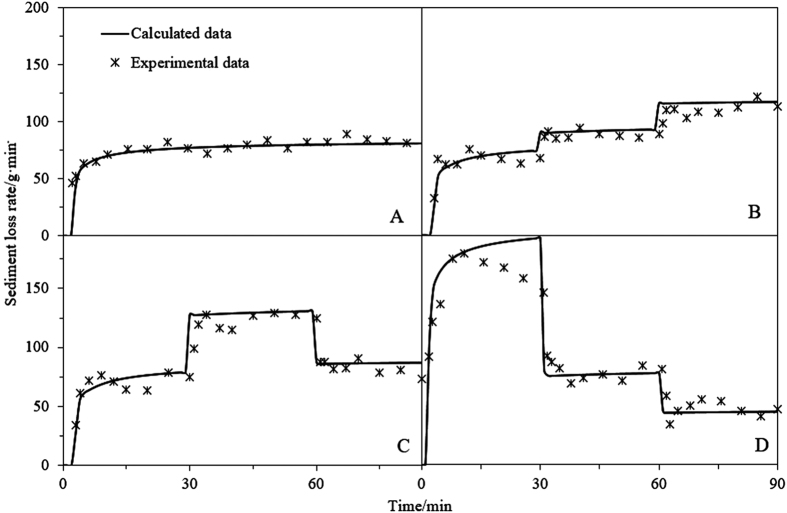
Comparison of the calculated and experimentally determined sediment yield rate. (**A**) is uniform-type, (**B**) is increasing-type, (**C**) is increasing- decreasing -type and (**D**) is decreasing-type.

Figure 2 and Supplementary Figure S1 show the sediment yield associated with each of the four rain pattern conditions. The variation of rainfall intensity during rainfall had significant effect on the process of sediment yield. For the same rain intensity, the sediment yield rate during the initial stages of rainfall is higher than that occurring in later stages. During continuous rainfall, the soil may become compacted by raindrop strikes, thereby resulting in crust formation and, consequently, a decrease in erosion. The sediment yield rate associated with the early stage of pattern D is significantly higher than that of the other patterns. This is attributed to the high rain intensity that results in a higher capacity of raindrop strikes and runoff scour than those occurring at low intensity. A total sediment yield of 5.83, 7.81, 8.10, and 8.73 kg was obtained for patterns A, B, C, and D, respectively. The variance analysis showed (see Table 1) that the effect of rainfall patterns on total sediment yield is very significant. Therefore, variations in the rainfall intensity have a significant effect on the total sediment yield, and early peaks in rainfall may lead to the most severe erosion.

Figure 2 shows the results determined from Equation (21). The calibration constant of runoff (*a*), the calibration constant of splash (*b*), RMSE and R^2^ are listed in Table 3. The calculated data concur with the measured values and, hence, the model can be used to predict the process of sediment transport associated with different rainfall patterns. As Table 3 shows, the calibration constants (*a* and *b*) vary with the rain period. The former (*a*) reflects the capacity for runoff scour, and the latter (*b*) reflects the response to raindrop strikes. These values (*a* and *b*) may increase and decrease, respectively, with increasing rainfall intensity indicating that runoff erosion may increase with increasing rain intensity, whereas raindrop splash may be subdued.

### Nutrients

**Figure 3:**
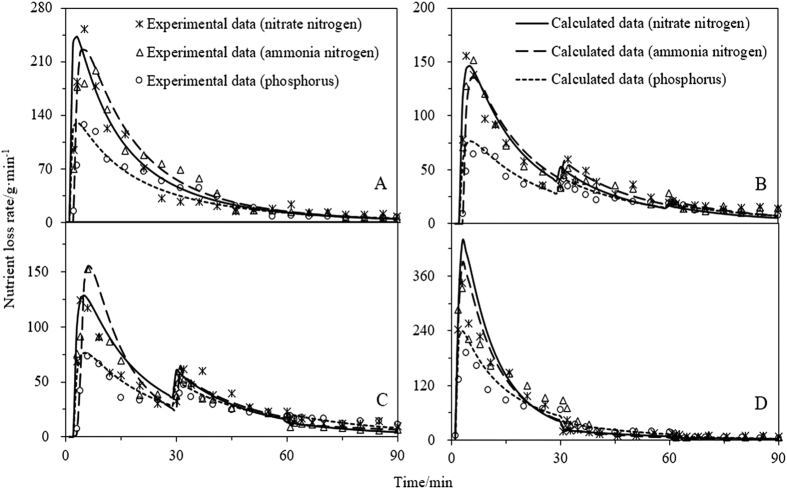
Comparison of the calculated and experimentally determined nutrient loss rate. (**A**) is uniform-type, (**B**) is increasing-type, (**C**) is increasing- decreasing -type and (**D**) is decreasing-type.

The nutrient loss associated with each of the four rainfall patterns is shown in Fig. 3 and Supplementary Fig. S2. Each change in rain intensity may result in upward or downward mutation of the nutrient loss rate; the direction of mutation depends on the direction of change in the rainfall intensity. For three types of nutrients, the nutrient loss rate in the initial stage of pattern D is significantly higher than those of the other patterns, owing possibly to a more severe interaction with surface soil. In fact, at high rain intensity, solute in the surface soil may be released into the surface runoff more rapidly than at low intensity; hence, higher rainfall intensity during the initial stages can lead to a higher rate of nutrient loss. Total losses of 0.58, 0.83, 0.91, and 0.99 g; 0.33, 0.35, 0.42, and 0.54 g; 0.23, 0.29, 0.32, and 0.38 g were determined for nitrate nitrogen, ammonia nitrogen, and phosphorous, respectively. The variance analysis of nutrient losses in different rainfall pattern conditions were listed in Table 1, the results showed that rainfall patterns have an evident effect on total nutrient loss. Compared to the other patterns, pattern D may result in more severe nutrient loss.

In addition, the rain intensity has a significant effect on the value of the parameter, *k* (obtained via curve fitting), which increases with increasing rain intensity (see Table 4). This indicates that high rain intensity may increase the rate of solute transfer from the surface soil to the surface runoff, thereby leading to increased nutrient loss. The degree of agreement between the calculated and the experimental data was quantified via RMSE and R^2^ (Table 4). The results indicate that the mathematic model of runoff solute transport provides an accurate description of the nutrient loss associated with different rainfall patterns.

## Discussion and Conclusion

Equations describing the sediment yield rate and nutrient loss rate were formulated in this study. To easily obtain an analytical solution, the sediment concentration and nutrient concentration in the runoff water were considered uniform. A similar assumption was used by Gao^37^ to calculate the chemical transport of nutrients from the soil to the runoff. During the simulated rainfall experiments, four rain patterns were applied in order to determine the effect of rain pattern on soil erosion and nutrient loss.

The change in rainfall intensity may have a significant impact on the process of runoff generation, but only a slight effect on the total amount of runoff. This is attributed to the fact that, in the initial stage, the infiltration capacity of the soil is higher than the rainfall intensity. In general, during the rain process, the rainfall patterns exhibited almost the same soil infiltration capacity and, hence, the total infiltration associated with these patterns was almost identical. However, the beginning of runoff varies with the pattern, i.e., the beginning time decreases with increasing intensity in the initial stage of rainfall. Frauenfeld^16^ obtained similar results in an investigation of rainfall-intensity effects on runoff. The simulations of the runoff model in this study indicate that the constant (*c*) decreases with increasing rain intensity and the value of *c* in the initial rain period is lower than that of subsequent stages.

The variation in rainfall intensity in a rain event had a significant effect on the sediment yield process and the total amount of sediment. In the initial stages of rainfall, soil erosion results mainly from raindrop strikes, which increase in strength with increasing intensity of the rainfall. In subsequent stages, the gradually increasing runoff rate may lead to severe soil erosion, and increasing rainfall intensity results in a sharp increase in the runoff rate. High rainfall intensity in the initial stages, and a combination of runoff scour and raindrop strikes result in total erosion of the soil, and a considerably higher sediment yield than that occurring at low rainfall intensity. Therefore, high-intensity rainfall in the initial stages may lead to the most severe erosion. This differs from the result of Flanagan^15^ who determined the effect of storm patterns on erosion. Furthermore, simulations of the sediment yield rate model revealed that the value of the calibration constant of splash erosion (*a*) and runoff erosion (*b*) may increase and decrease, respectively, with increasing rainfall intensity; *a* reflects the ability for runoff scour, and *b* reflects the response to raindrop strikes. This suggests that runoff erosion increases with increasing rainfall intensity, but raindrop splash may be subdued.

The effect of rainfall pattern on nutrient loss has rarely been investigated. However, the results of these studies indicate that the rain pattern has a significant effect on this loss. Most of the nutrients in the soil surface may be washed away by infiltration water prior to the runoff yield. Therefore, the infiltration capacity of the soil may have a considerable impact on the nutrient concentration of the runoff water. The four plots considered in this study had similar soil properties and, hence, the infiltration capacity of these plots was considered the same. Compared with low-intensity rainfall, high-intensity rainfall interacts more severely with the soil surface and therefore, significantly higher amounts of nutrient near the soil surface may be transported into the runoff water. The nutrient concentration near the soil surface will decrease rapidly and, hence, high-intensity rainfall during the late stages may have only a modest effect on the nutrient concentration of the runoff water; in other words, compared with the other types, the early peaking type results in a higher rate of nutrient loss. In this study, simulations of the nutrient model revealed that the convective mass transfer coefficient (*k*) increases with increasing rainfall intensity.

The mathematical model developed in this study can accurately predict sediment yield and nutrient loss because of the calculated data concur with the measured data. However, all the simulation experiments were based on bare land. The practicability of the model for vegetation cover conditions need to be further evaluated.

## Materials and Methods

### Site description

The experiments were conducted at the Changwu State Key Agro-Ecological Experimental Station of the Loess Plateau (35°14′N, 107°41′E), which is located in the rain-fed cropping region of the Loess Plateau in China. The elevation is 1000 m above sea level, and mean annual temperature and annual precipitation were 9.1 °C and 473 mm, respectively. The climate of the region can be described as a sub-humid continental monsoon. However, the distribution of precipitation exhibits significant seasonal variation. The groundwater level of the station reaches a depth of 60 m^38^.

### Experimental method

**Figure 4:**
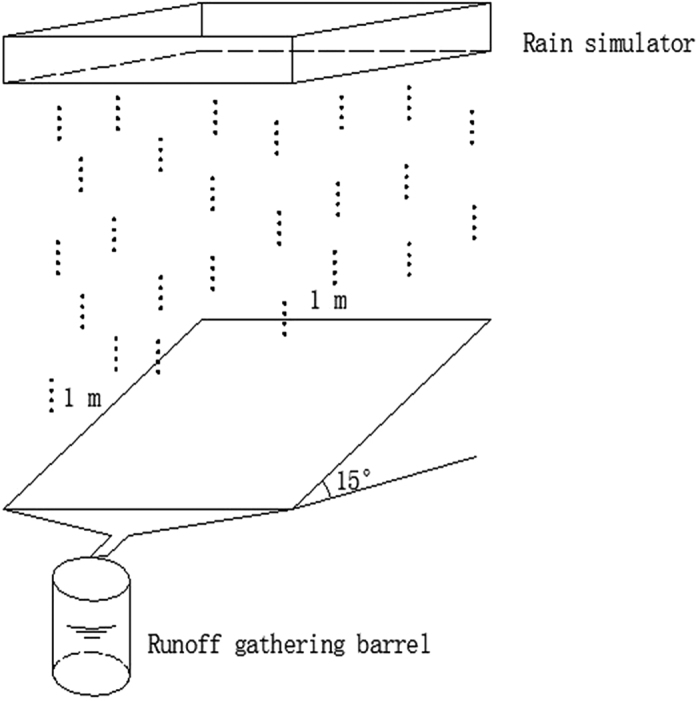
Simulation rainfall system.

Rainfall erosion was investigated on four runoff plots (1 × 1 m^2^) established on a hillslope with a gradient of 15°. Each 90-min simulated rainfall event was performed by using a syringe-driven rain simulator (see Fig. 4). Furthermore, the rain height was 1 m from the soil surface and rainfall intensities of 100–160 mm/h were considered. The intensity was varied by adjusting the water level in the sink of the rain simulator. The mechanical composition of the soil (bulk density: 1.41 g/cm^3^) surface was measured using a laser particle size analyzer. These measurements revealed a clay, silt, and sand content of 3.47%, 92.26%, and 4.27%, respectively. The silt clay loam shown in the soil of erosion plots is categorized in accordance with the international classification.

Four rainfall patterns were considered during the experiment, namely: A (uniform-type: 130-130-130 mm/h), B (increasing-type: 100-130-160 mm/h), C (increasing-decreasing-type: 100-160-130 mm/h), and D (decreasing-type: 160-130-100 mm/h). Each rainfall intensity has a corresponding water level in the sink of the rain simulator. The water level in the sink could be increased or decreased by fast supplying water or drainage when the rainfall intensity need to be changed. Thus, the variation of rainfall intensity could be implemented during rainfall period. All the treatments were performed in triplicate. Each rainfall pattern was divided into three 30-min stages. In addition, the runoff, sediment, and nutrient were collected in a 30-cm-diameter runoff gathering barrel placed at the bottom of the slope. The runoff generated was determined by measuring the water level in the barrel. Water samples of the runoff were collected at predefined times from the outlet of each plot. The amount of sediment was determined by drying the samples, and the concentration of nutrients in the runoff was evaluated via ultraviolet and visible spectroscopy.

### Theoretical analysis

#### Governing equations

The runoff process during rainfall can be described by a mass conservation equation, which is given as:


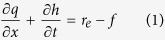


where *q(x, t*) is unit discharge (cm^2^/min), *h(x, t*) is runoff depth (cm), *x* is the distance (cm) along the overland flow plane, *t* is time (min), *f(t*) is infiltration rate (cm/min), *r*_*e*_ is the actual accepted rainfall intensity of unit soil surface (cm/min).

Assuming that the change in runoff depth is proportional to the excess rainfall^39^ and therefore:


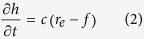


where *c* is a constant. Substitution of Equation (2) into Equation (1) yields the simplified kinematic wave equation:


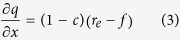


The boundary and initial conditions for Equation (3) are:


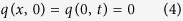


The corresponding sediment transport equation can be expressed as ref. 40:


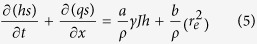


where *s(x, t*) is the sediment concentration in runoff water (g/cm^3^), *ρ* is soil bulk density (g/cm^3^), *γ* is water bulk density (g/cm^3^), *J* is hydraulic slope, *a* is the calibration constant of runoff erosion, *b* is the calibration constant of splash erosion.

Combining Equations (1) and (5) yields:


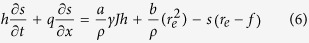


The boundary and initial conditions for Equation (6) are:


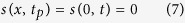


where *t*_*p*_ is time of ponding (i.e., the time when *r*_*e*_ = *f(t*_*p*_)).

The solute in the soil surface layer is transferred to the overland flow by a rate-limited mass transfer process. Furthermore, a mass conservation equation can be used to describe runoff solute transport via surface runoff toward the slope outlet^41^:


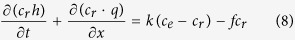


where *c*_*r*_(*x, t*) is the solute concentration (mg/L) in runoff water, *c*_*e*_(*t*) is the solute concentration (mg/L) at the soil surface; the value of *c*_*e*_(*t*) is time dependent owing to solute depletion by transfer to the surface runoff and downward displacement with infiltration water (i.e., when infiltration occurs); *k* is convective mass transfer coefficient (cm/min), the value of *k* depends on the specific solute involved in the process, physical characteristics of the soil surface, rain intensity, and duration.

Combining Equations (1) and (8) yields:


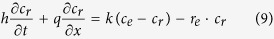


The boundary and initial conditions for Equation (9) are:


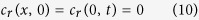


Solute transport in the soil profile is controlled by infiltration and diffusion and can be described by the convective-dispersion equation:


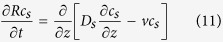


where *c*_*s*_(*z, t*), *R, v*, and *D*_*s*_ are the solute concentration (mg/L) in the soil profile, retardation factor, average pore-water velocity (cm/min), and dispersivity coefficient (cm/min) of solute in the soil, respectively.

The vertical direction of soil profile was considered as a semi-finite long condition, and the boundary and initial conditions for Equation (11) are:


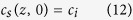



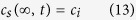



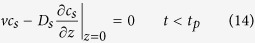



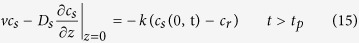


where c_*i*_ is the initial soil solute concentration (mg/L).

#### Solution of the surface runoff equation

The infiltration rate at the soil surface can be determined from Philip’s^42^ equation, where the process of infiltration occurring during rainfall is taken into consideration. Runoff is prevented when the soil infiltration capacity is higher than the rainfall intensity; the infiltration rate is equal to the rainfall intensity during this period. Runoff begins when the infiltration rate exceeds the rainfall intensity. Yang^28^ used the modified Philip equation to describe the process of infiltration during rainfall, and subsequently solved Equation (3).

The infiltration rate can be expreseed as:


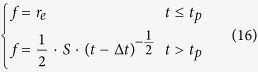


where *S* is adsorptivity (cm/min^0.5^) and 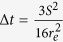.

The unit discharge at the outlet can be expressed as:


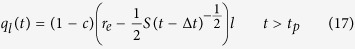


where *q*_*l*_ is the unit discharge (cm^2^/min) at the outlet, *l* is slope length (cm).

Combining Manning’s formula and Equation (16) yields the runoff depth at the outlet:


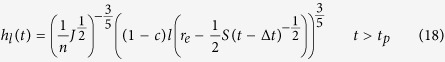


where *n* and *J* are the Manning’s roughness coefficient (s/m^1/3^) and hydraulic gradient, respectively.

#### Solution of the sediment transport equation

Assuming that the runoff water has a uniform concentration of sediment along the hillslope, substitution of Equation (18) into (6), yields the mass conservation equation for sediment at the outlet:


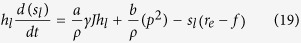


where *s*_*l*_is the runoff sediment concentration (g/cm^3^) at the outlet.

Based on the initial condition for Equation (8), the runoff sediment concentration at the outlet can be expressed as:


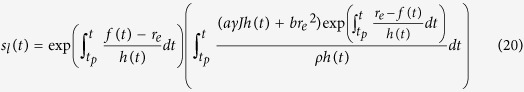


The sediment yield rate at the outlet can be calculated from:


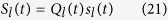


where *S*_*l*_(*t*) is the sediment yield rate (g/min) at the outlet, *Q*_*l*_(t) is the discharge at the outlet (cm^3^/min).

#### Solution of the advection diffusion equation

For simplicity, the retardation factor, *R*, the average pore-water velocity, *v*, and dispersivity coefficient, *Ds*, were assumed to be constant; the solute concentration in the runoff is significantly lower than that in the near-surface soil and, hence, the runoff concentration (*c*_*r*_) was neglected^43^. Thus, the solution of Equation (11), subject to boundary and initial conditions, corresponding to the solute concentration at the soil surface can be expressed as:


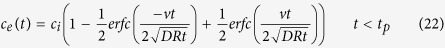



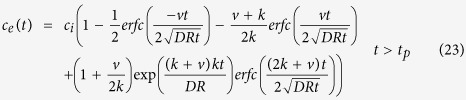


#### Solution of the runoff solute transport equation

Assuming that the runoff water has a uniform concentration of solute along the hillslope. Thus, the Equation (9) was simplified since the value of ∂*c*_*r*_/∂*x* in the equation is 0. Substitution of Equation (18) into (9) yields the mass conservation equation for runoff solute at the outlet:


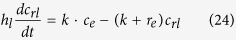


where *c*_*rl*_(*t*) is the solute concentration of runoff water (mg/L) at the outlet.

Based on the initial condition associated with Equation (11), the runoff solute concentration at the outlet can be expressed as:


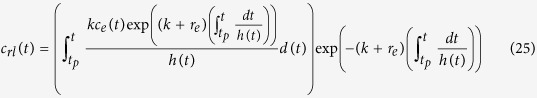


The solute loss rate in the runoff can be calculated from^44^:


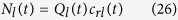


where *N*_*l*_(*t*) is the solute loss rate (mg/min) in the runoff at the outlet.

#### Relevant parameters

**Figure 5:**
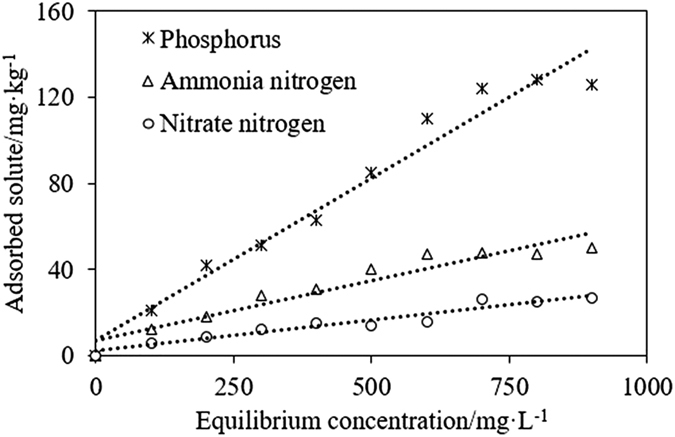
The adsorption isotherm of three types of solute.

Tao^45^ obtained a Manning’s roughness coefficient of *n* = 0.017 s/m^1/3^ for the soil used in this experiment. Furthermore, Yang^46^ obtained a dispersivity coefficient of *D*_*s*_ = 0.14 cm/min, for the solute transport parameters of a soil similar to the one considered in the present study. In this study, the average pore-water velocity (*v*) was considered the stable infiltration rate. The retardation factor, *R*, can be expressed as:


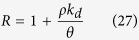


where *θ* is water content of the soil profile (cm^3^/cm^3^). For simplicity, *θ* was assumed equal to the saturated water content; *k*_*d*_ is solute distribution coefficient (L/kg) of solute adsorbed to the solid phase. Figure 5 shows the adsorption isotherms associated with three types of solute (nitrate nitrogen, ammonia nitrogen, and phosphorus). For the mathematical simplicity, linear forms of the isotherms models are also widely adopted to determine the isotherm parameters^47^. The corresponding isothermal adsorption equations are given as follows^48^:


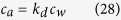


where *c*_*a*_ is the solute adsorbed on soil (mg/kg) at equilibrium; *c*_*w*_ is the equilibrium concentration (mg/L), which is the solute concentration when sorption-desorption equilibrium (evaluated by ultraviolet and visible spectroscopy). Adsorption coefficients of 0.16, 0.07, and 0.03 were determined for phosphorus, ammonia nitrogen, and nitrate nitrogen, respectively. These parameters are all listed in Table 5.

The adsorptivity (*S*) was obtained from fitting infiltration curves by Equation (16). The constant (c) was obtained from fitting runoff curves by Equation (17). When the value of *S* and *c*, the calibration constant of runoff erosion (*a*) and calibration constant of splash erosion (*b*) can be obtained from fitting sediment yield curves by Equation (20). And the rate of soil water ejection into the runoff (*k*) also can be obtained from fitting nutrient loss curves by Equation (25).

## Additional Information

**How to cite this article:** Tao, W. *et al*. Mathematical model of sediment and solute transport along slope land in different rainfall pattern conditions. *Sci. Rep.* **7**, 44082; doi: 10.1038/srep44082 (2017).

**Publisher's note:** Springer Nature remains neutral with regard to jurisdictional claims in published maps and institutional affiliations.

## Supplementary Material

Supplementary Information

## Figures and Tables

**Table 1:** Variance analysis of experimentdal data.

Object	SS_A_	SS_E_	v_A_	v_E_	MS_A_	MS_E_	F	F_0.05_	Significance
Runoff	3.26	28.65	3	8	1.09	3.58	0.30	4.07	−
Sediment	14.11	0.24			4.70	0.03	154.58		+
Nitrate nitrogen	0.28	0.07			0.09	0.01	11.18		+
Ammonia nitrogen	0.08	0.03			0.03	0.003	7.81		+
Phophorus	0.034	0.02			0.01	0.002	4.43		+

SS_A_ and SS_E_ are the intraclass variance and interclass variance, v_A_ and v_E_ are the freedom degree of influence factor and error, MS_A_ and MS_E_ are the mean variance of influence factor and error, F is the test statistics, F_0.05_ is the critical test statistics when the value of significance level is equal to 0.05, +shows significant, −shows non-significant.

**Table 2:** Values of *c*, RMSE, and R^2^ for the different rain patterns with fitting of the experimental data.

Rainfall patterns	*c*			*S/*cm·min^−0.5^	RMSE	R^2^
	Period1	Period2	Period3			
A		0.013		0.46	1.30	0.87
B	0.035	0.014	0.007		1.33	0.94
C	0.035	0.005	0.017		1.26	0.91
D	0.002	0.012	0.042		1.45	0.83

**Table 3:** Values of a, b, RMSE, and R^2^ for the different rain patterns with fitting of the experimental data.

Rainfall patterns	a			b			RMSE	R^2^
	Period1	Period2	Period3	Period1	Period2	Period3		
A		0.07			0.06		5.02	0.87
B	0.07	0.08	0.09	0.12	0.07	0.05	7.10	0.88
C	0.07	0.09	0.06	0.13	0.06	0.07	8.81	0.87
D	0.08	0.07	0.06	0.11	0.06	0.06	20.22	0.81

**Table 4:** Values of *k*, RMSE, and R^2^ for the different rain patterns with fitting of the experimental data.

Rainfall patterns		*k*			RMSE	R^2^
		Period1	Period2	Period3		
Nitrate nitrogen	A		0.07		26.43	0.85
	B	0.06	0.09	0.12	11.22	0.91
	C	0.05	0.10	0.08	11.90	0.85
	D	0.12	0.07	0.05	39.19	0.83
Ammonia nitrogen	A		0.07		26.95	0.81
	B	0.05	0.08	0.12	16.71	0.80
	C	0.05	0.09	0.06	15.19	0.81
	D	0.1	0.07	0.05	38.21	0.84
Phosphorus	A		0.05		12.74	0.88
	B	0.04	0.07	0.11	6.22	0.87
	C	0.04	0.08	0.06	7.47	0.79
	D	0.08	0.06	0.04	22.45	0.80

**Table 5:** Parameters used in the mathematical model.

Parameters	Value
Saturated water content, *θ*_*s*_ (cm^3^ · cm^−3^)	0.51
Bulk density, *ρ* (g · cm^−3^)	1.41
Initial soil solution concentration, *c*_*i*_(mg/L)	291( 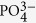 -P), 487( 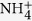 -N), 504( 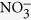 -N)
Saturated hydraulic conductivity, *k*_*s*_ (cm/min)	0.012
Manning’s roughness coefficient, *n* (s · m^−1/3^)	0.017
Dispersivity coefficient, Ds (cm · min^−1^)	0.14
Retardation factor, R = 1 + 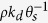	1.44( 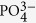 -P), 1.19( 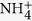 -N), 1.08( 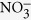 -N)
Pore-water velocity, *v* (cm · min^−1^)	0.03
