# Supplementary material for: Mathematical model of sediment and solute transport along slope land in different rainfall pattern conditions
Source: Sci Rep. 2017 Mar 8;7:44082. doi: 10.1038/srep44082 (PMC5341045; doi:10.1038/srep44082)
Supplement: Supplementary Information [file srep44082-s1.pdf]

Mathematical model of sediment and solute transport along slope land in different rainfall pattern conditions

Wanghai Tao[1], Junhu Wu[1], & Quanjiu Wang[1,2,*]

[1]State Key Laboratory Base of Eco-hydraulic Engineering in Arid Area (Xi'an University of Technology), Xi'an, 710048, China

[2] State Key Laboratory of Soil Erosion and Dryland Farming on the Loess Plateau, Institute of Soil and Water Conservation, Northwest A＆F University, Xi'an, 712100, China

**Corresponding to: Wang, Q. J.* (wquanjiu@163.com)

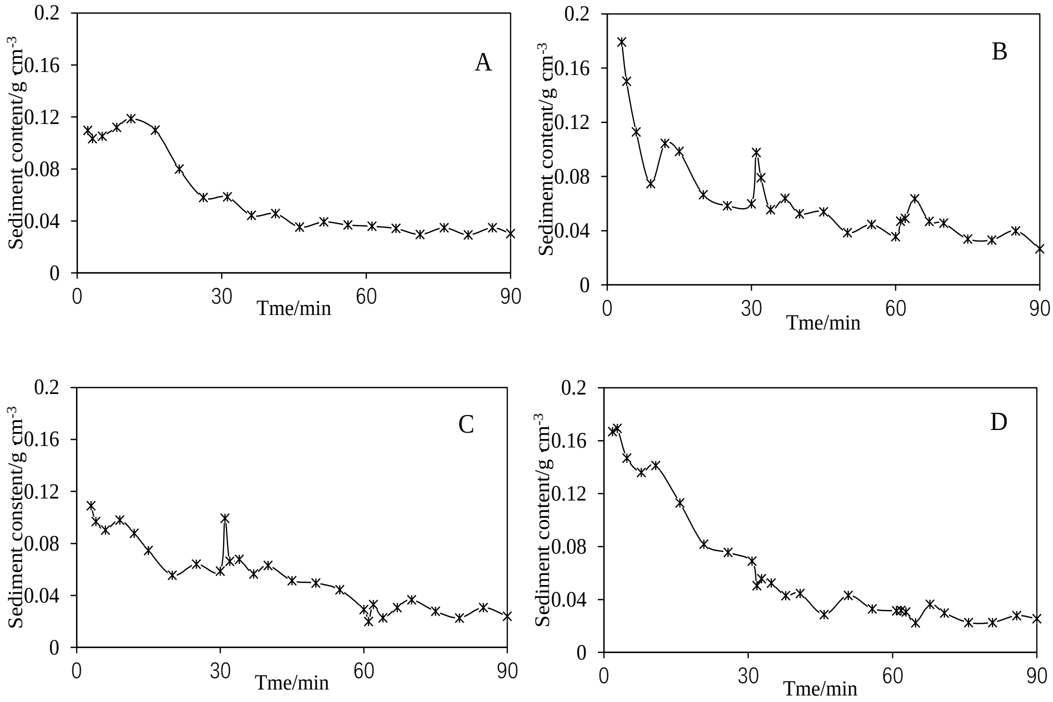

Figure S1. Sediment content of surface flow in different rainfall patterns. A, B, C and D are constant pattern, latter peaking pattern, mid peaking pattern and early peaking pattern, respectively.

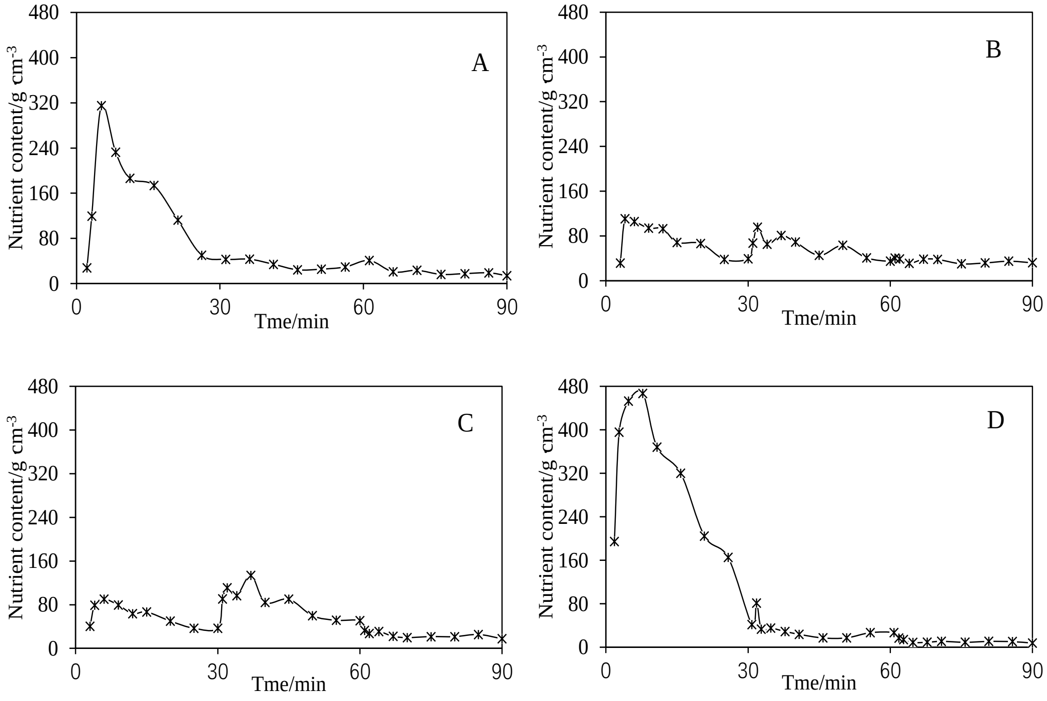

Figure S2. Nitrate nitrogen content of surface flow in different rainfall patterns.

A, B, C and D are constant pattern, latter peaking pattern, mid peaking pattern and early peaking pattern, respectively.

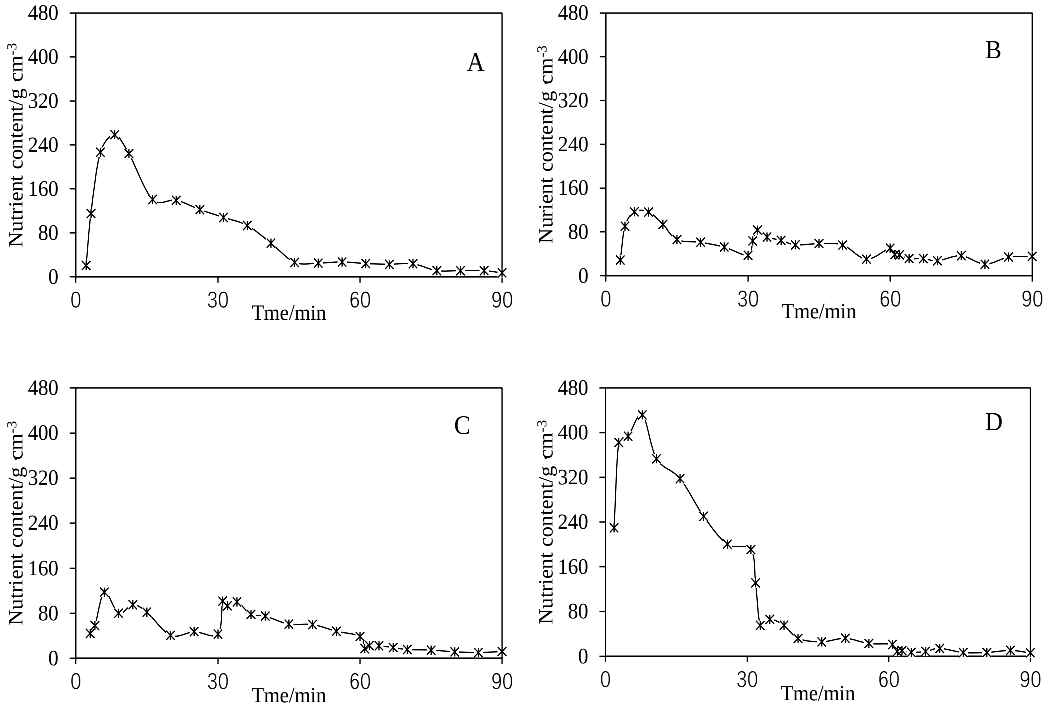

Figure S3. Ammonia nitrogen content of surface flow in different rainfall patterns.

a, b, c and d are constant pattern, latter peaking pattern, mid peaking pattern and early peaking pattern, respectively.

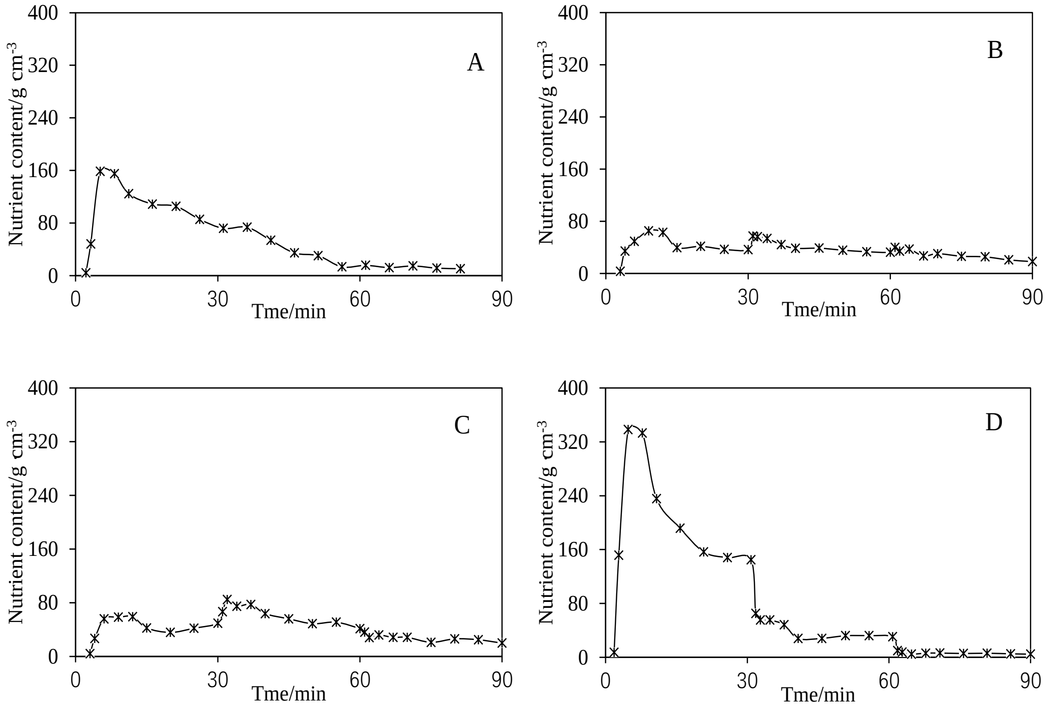

Figure S4. Phosphorus content of surface flow in different rainfall patterns. A, B, C and D are constant pattern, latter peaking pattern, mid peaking pattern and early peaking pattern, respectively.
